# Pathways to diagnosis of non-small cell lung cancer: a descriptive cohort study

**DOI:** 10.1038/s41533-018-0113-7

**Published:** 2019-02-08

**Authors:** Stuart Purdie, Nicola Creighton, Kahren Maree White, Deborah Baker, Dan Ewald, Chee Khoon Lee, Alison Lyon, Johnathan Man, David Michail, Alexis Andrew Miller, Lawrence Tan, David Currow, Jane M. Young

**Affiliations:** 1 Cancer Institute NSW, Sydney, NSW Australia; 2 North Coast Primary Health Network, Ballina, NSW Australia; 3 University Centre for Rural Health, Lismore, NSW Australia; 4 Cancer Care Centre, St George Hospital, Sydney, NSW Australia; 5 NHMRC Clinical Trials Centre, University of Sydney, Sydney, NSW Australia; 6 School of Medicine, Western Sydney University, Sydney, NSW Australia; 7 Crown Princess Mary Cancer Centre, Westmead Hospital, Sydney, NSW Australia; 8 Illawarra Cancer Care Centre, Wollongong Hospital, Wollongong, NSW Australia; 9 Centre for Oncology Informatics, University of Wollongong, Gwynneville, NSW Australia; 10 School of Public Health, University of Sydney, Sydney, NSW Australia; 11 Surgical Outcomes Research Centre, Sydney Local Health District, Sydney, NSW Australia

## Abstract

Little has been published on the diagnostic and referral pathway for lung cancer in Australia. This study set out to quantify general practitioner (GP) and lung specialist attendance and diagnostic imaging in the lead-up to a diagnosis of non-small cell lung cancer (NSCLC) and identify common pathways to diagnosis in New South Wales (NSW), Australia. We used linked health data for participants of the 45 and Up Study (a NSW population-based cohort study) diagnosed with NSCLC between 2006 and 2012. Our main outcome measures were GP and specialist attendances, X-rays and computed tomography (CT) scans of the chest and lung cancer-related hospital admissions. Among our study cohort (*N* = 894), 60% (*n* = 536) had ≥4 GP attendances in the 3 months prior to diagnosis of NSCLC, 56% (*n* = 505) had GP-ordered imaging (chest X-ray or CT scan), 39% (*N* = 349) attended a respiratory physician and 11% (*N* = 102) attended a cardiothoracic surgeon. The two most common pathways to diagnosis, accounting for one in three people, included GP and lung specialist (respiratory physician or cardiothoracic surgeon) involvement. Overall, 25% of people (*n* = 223) had an emergency hospital admission. For 14% of people (*N* = 129), an emergency hospital admission was the only event identified on the pathway to diagnosis. We found little effect of remoteness of residence on access to services. This study identified a substantial proportion of people with NSCLC being diagnosed in an emergency setting. Further research is needed to establish whether there were barriers to the timely diagnosis of these cases.

## Lung cancer: The pathways to diagnosis

Examining events leading to the diagnosis of non-small cell lung cancer (NSCLC) in Australia yields insights to guide further research and perhaps improve the pathways to diagnosis. NSCLC is by far the most common form of lung cancer. Researchers, led by the Cancer Institute New South Wales, investigated clinical care contacts leading to diagnosis, using a descriptive cohort study of 894 patients diagnosed between 2006 and 2012. The researchers quantified contact with GPs and lung specialists, hospital admissions and diagnostic imaging procedures. Living in remote locations had little influence on access to services. More than half of the patients did not see a lung specialist during the pathway to diagnosis, while a quarter received their diagnosis in an emergency setting. Further research should investigate whether there are barriers preventing timely diagnosis.

## Introduction

Lung cancer kills more people than any other cancer in Australia.^1^ Five-year survival has improved since the 1980s but remains poor (16% for 2009–2013).^1^ Evidence-based treatment is critical to further improve outcomes for people with lung cancer. Numerous studies examining treatment pathways for people with lung cancer in Australia have identified gaps in optimal treatment delivery.^2–8^ While there are studies that have focussed on the pathway from the point of specialist referral and treatment planning onwards^4-9^, there are fewer studies on the primary care, diagnostic and referral pathways to the point of specialist contact.^10,11^ To our knowledge, no Australian study has examined the use of diagnostic imaging and specialist referral in the lead-up to lung cancer diagnosis.

Although lung cancer is one of the most common cancers in Australia (43 new cases per 100,000 people per year^1^), it will be encountered rarely by an individual general practitioner (GP) and diagnosis can be challenging. The early symptoms of lung cancer, such as weight loss, a new or changing cough, shortness of breath and chest pain, are often non-specific. The absence of symptoms for many people until disease is advanced makes early diagnosis challenging. Haemoptysis, a “red flag” symptom, only occurs in around one fifth of people presenting with lung cancer.^12,13^ Most people require imaging beyond a simple chest X-ray to confirm the diagnosis of lung cancer.^14^

People with suspected lung cancer should be referred for specialist assessment, including obtaining a tissue diagnosis and oversight by a multi-disciplinary team charged with developing a consensus-based treatment plan.^14,15^ New South Wales (NSW) studies found that a lung cancer multidisciplinary team (MDT) increased the use of treatment,^16^ while reduced access to specialist care may account for poorer outcomes for people in rural settings.^3,17^

In this study, we aimed to quantify the use and pattern of GP attendances, specialist attendances and diagnostic imaging in a community/outpatient setting in the lead-up to diagnosis and identify common diagnostic pathways of NSW residents with non-small cell lung cancer (NSCLC). A secondary aim was to measure health service use by remoteness of residence.

## Results

A total of 1235 people were diagnosed with NSCLC between enrolment into the 45 and Up Study (February 2006) and 31 December 2012. There were 894 people in the study cohort (Table 1) after excluding cases notified only by death certificate (*n* = 34), people with another cancer case diagnosed between 2000 and 2012 (*n* = 251) and cases with an estimated diagnosis date (*n* = 56).

**Table 1:** Demographic and tumour characteristics of study participants with non-small cell lung cancer

Characteristic	*n*	% (*N* = 894)
Age at diagnosis, years		
45–64	195	21.8
65–79	453	50.7
80+	246	27.5
Sex		
Female	385	43.1
Male	509	56.9
Remoteness of residence		
Major cities	477	53.4
Inner regional	314	35.1
Outer regional/remote	103	11.5
Area-based socioeconomic status		
Least disadvantaged quintile	137	15.3
Quintile 2	147	16.4
Quintile 3	176	19.7
Quintile 4	256	28.6
Most disadvantaged quintile	178	19.9
Smoking status at enrolment^a^		
Current smoker	217	24.3
Ex-smoker	524	58.6
Never smoked regularly	150	16.8
Extent of disease at diagnosis		
Localised	173	19.4
Regional	204	22.8
Distant	417	46.6
Unknown	100	11.2
Best method of diagnosis		
Histopathologically verified	642	71.8
Cytology	169	18.9
Clinical/imaging/biochemistry	83	9.3
Charlson comorbidity score		
0	682	76.3
1	130	14.5
≥2	82	9.2

^a^Excludes people with unknown smoking status

### Use of health services in the lead-up to diagnosis

**Fig. 1:**
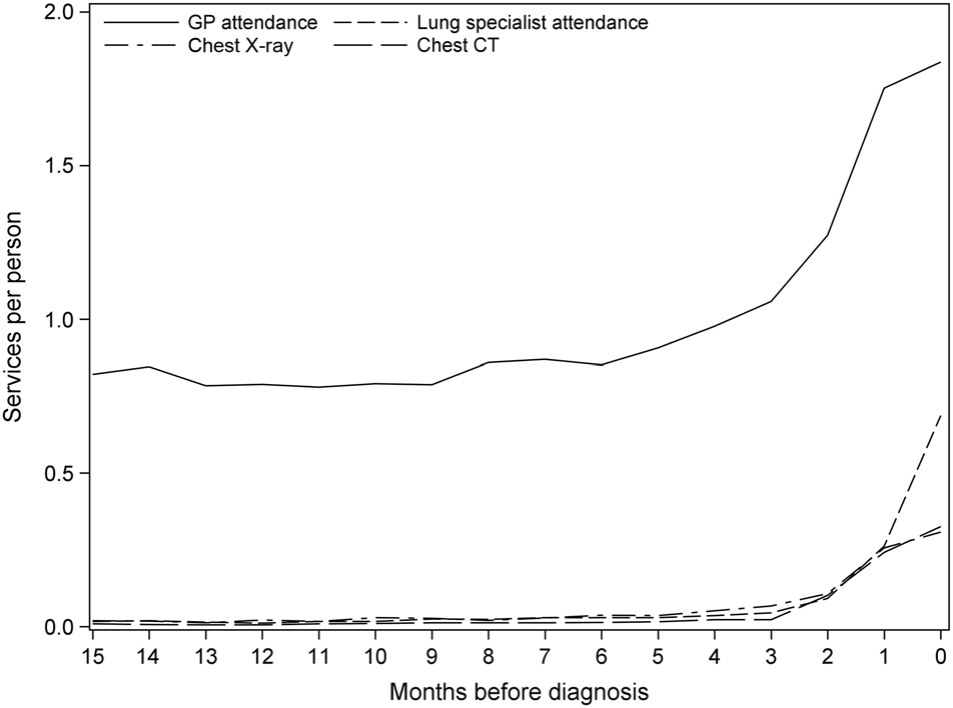
GP attendances, lung specialist attendances, chest X-rays and chest CT scans per person in the 15 months before non-small cell lung cancer diagnosis

The rate of GP attendance was <1 per person 15 months prior to NSCLC diagnosis, increasing gradually from 6 months prior to diagnosis (Fig. 1) before reaching a peak of just under two per person in the month of diagnosis. Rapid increases in the number of chest X-rays, chest computed tomography (CT) scans and lung specialist attendances in a community setting coincided with the rapid increase in GP attendances from 2 months before diagnosis.

In the 3 months to diagnosis, 93% of people attended a GP, with 60% attending ≥4 times; 39% attended a respiratory physician; 11% attended a cardiothoracic surgeon; while <10% attended a medical or radiation oncologist, general physician or general surgeon (Table 2). Just under half (46%) of people had a diagnostic procedure (bronchoscopy or biopsy) recorded. Just over half had a chest X-ray (53%) or chest CT scan (59%) in a community setting, with most imaging ordered by a GP. Around 40% of people had both a chest X-ray and CT scan and almost a third of people did not have a record of either (Table 3). We could not determine how many people saw a lung specialist or had chest imaging during an inpatient admission.

**Table 2:** Use of selected health services in the 3 months to diagnosis of non-small cell lung cancer by remoteness of residence

Service	Number of services	Major cities (*N* = 477)		Inner regional (*N* = 314)		Outer regional/remote (*N* = 103)		Total (*N* = 894)		*χ*^2^ test
		*n*	%	*n*	%	*n*	%	*n*	%	
GP attendance	0	35	7.3	17	5.4	7	6.8	59	6.6	*χ*^2^(8) = 15.66, *P* = 0.05
	1–3	164	34.4	102	32.5	33	32.0	299	33.4	
	4–6	162	34.0	133	42.4	49	47.6	344	38.5	
	7-9	79	16.6	45	14.3	6	5.8	130	14.5	
	>9	37	7.8	17	5.4	8	7.8	62	6.9	
Respiratory physician attendance	≥1	196	41.1	117	37.3	36	35.0	349	39.0	*χ*^2^(2) = 1.98, *P* = 0.37
Cardiothoracic surgeon attendance	≥1	46	9.6	44	14.0	12	11.7	102	11.4	*χ*^2^(2) = 3.58, *P* = 0.17
Medical oncologist attendance	≥1	24	5.0	14	4.5	7	6.8	45	5.0	*χ*^2^(2) = 0.89, *P* = 0.64
Radiation oncologist attendance	≥1	32	6.7	20	6.4	9	8.7	61	6.8	*χ*^2^(2) = 0.71, *P* = 0.70
General physician attendance	≥1	40	8.4	20	6.4	5	4.9	65	7.3	*χ*^2^(2) = 2.15, *P* = 0.34
General surgeon attendance	≥1	31	6.5	27	8.6	9	8.7	67	7.5	*χ*^2^(2) = 1.46, *P* = 0.48
Chest X-ray (outpatient)	≥1	251	52.6	174	55.4	49	47.6	474	53.0	*χ*^2^(2) = 1.98, *P* = 0.37
GP ordered^a^	≥1	196	41.1	150	47.8	38	36.9	384	43.0	*χ*^2^(2) = 5.19, *P* = 0.07
Specialist ordered^a^	≥1	74	15.5	33	10.5	12	11.7	119	13.3	*χ*^2^(2) = 4.39, *P* = 0.11
CT scan (outpatient)	≥1	280	58.7	189	60.2	62	60.2	531	59.4	*χ*^2^(2) = 0.21, *P* = 0.90
GP ordered^a^	≥1	200	41.9	148	47.1	48	46.6	396	44.3	*χ*^2^(2) = 2.33, *P* = 0.31
Specialist ordered^a^	≥1	93	19.5	45	14.3	15	14.6	153	17.1	*χ*^2^(2) = 4.10, *P* = 0.13
Diagnostic procedures (inpatient/outpatient)^b^	≥1	222	46.5	145	46.2	48	46.6	415	46.4	*χ*^2^(2) = 0.01, *P* = 0.99

^a^GP-ordered and specialist-ordered categories are not mutually exclusive: people can have both GP-ordered and specialist-ordered imaging

^b^Bronchoscopy or biopsy

**Table 3:** Number (%) of chest X-rays and chest CT scans in the 3 months to diagnosis of non-small cell lung cancer

Number of chest X-rays	Number of chest CT scans			Total (column %)
	0	1	2	
0	262 (29%)	148 (17%)	10 (1%)	420 (47%)
1	94 (11%)	284 (32%)	16 (2%)	394 (44%)
≥2	7 (1%)	65 (7%)	8 (1%)	80 (9%)
Total (row %)	363 (41%)	497 (56%)	34 (4%)	894 (100%)

Although there were some differences in distribution of number of GP attendances, the median and interquartile range of GP attendances were the same for all remoteness areas (median: 4, interquartile range: 3–6). Specialist attendances were similar across remoteness areas. Respiratory physician attendance was not substantially higher (*χ*^2^(2) = 1.98, *P* = 0.37) for those living in major cities (41%), compared with inner regional (37%) and outer regional/remote areas (35%).

### Pathways to diagnosis

The two most common pathways to diagnosis, accounting for 30% of people, included GP-ordered imaging and lung specialist attendance without an emergency hospital admission (Table 4). Pathways including a lung specialist attendance (*n* = 405, 45%) had a higher proportion of cases with a histopathologically confirmed diagnosis (77% vs 67%, *χ*^2^(1) = 11.0, *P* ≤ 0.001), a lower proportion of emergency hospital admissions (8% vs 39%, *χ*^2^(1) = 111.6, *P* < 0.001) and a lower proportion of lung cancers with distant spread (31% vs 59%, *χ*^2^(1) = 69.5, *P* < 0.001).

**Table 4:** Pathways to diagnosis of non-small cell lung cancer

Rank	GP-ordered imaging	Lung specialist attendance	Elective admission	Emergency admission	*n*	% (*N* = 894)
1	●	●	●		136	15.2
2	●	●			132	14.8
3				●	129	14.4
4	●				119	13.3
5					96	10.7
6		●			61	6.8
7	●			●	61	6.8
8			●		45	5.0
9		●	●		43	4.8
10	●		●		39	4.4
11	●	●		●	18	2.0
12		●		●	15	1.7

Note: GP attendance is not included in the pathways because we could not distinguish attendances for lung-related symptoms from unrelated attendances

The third most common pathway (*n* = 129, 14%) was an emergency hospital admission without GP-ordered imaging or lung specialist attendance in the community. Overall, 223 people (25%) had an emergency hospital admission. People with an emergency admission had a higher proportion of cancers with distant spread (67% vs 40%, *χ*^2^(1) = 50.8, *P* < 0.001) and a lower proportion without comorbidity (69% vs 79%, *χ*^2^(1) = 8.6, *P* < 0.014).

Of the 11% (*n* = 96) of people with no events of interest (pathway 5), 15 (16%) saw a general physician or surgeon and 11 (12%) saw a medical or radiation oncologist.

## Discussion

The use of community-based health services associated with lung cancer diagnosis increased rapidly in the 2 months before diagnosis of NSCLC. The key role that GPs play in the diagnosis of lung cancer is highlighted by the findings that in the 3 months to diagnosis 93% attended a GP with 60% of people attending a GP at least four times and a similar proportion having GP-ordered thoracic imaging.

The two most common pathways to diagnosis of NSCLC, accounting for one in three people diagnosed, involved GP and lung specialists in the community without an emergency hospital admission, suggesting patients presenting with less severe or more typical lung cancer symptoms being referred to appropriate specialists. Nevertheless, fewer than half (45%) of people attended a lung specialist (respiratory physician or cardiothoracic surgeon) in the lead-up to diagnosis, which is similar to an earlier NSW study that reported only 53% of people with lung cancer saw a respiratory physician at initial presentation.^5^ Reasons for not seeing a lung specialist in the community before a diagnosis of NSCLC could include the sudden onset of severe symptoms requiring emergency presentation to hospital (e.g. haemoptysis), referral to a non-lung specialist (e.g. an oncologist or a general physician), referral to a specialist for the presenting symptoms of distant metastases (e.g. a neurologist) or an incidental finding of lung cancer while undergoing investigation for another condition. An Australian study reported that 23% of people with lung cancer were diagnosed incidentally.^7^ Incidental findings will account for some people diagnosed without GP-ordered imaging or lung specialist attendance in our study.

There were no substantial differences in patterns of GP and specialist attendances and chest imaging for people living in outer regional and remote areas compared with major cities. It appears that remoteness of residence alone is not a major barrier to seeing a lung specialist. However, we had no information on lung cancer MDT membership for the specialists, which may vary by remoteness. Further research is needed to determine whether referral to specialists who are active members of lung cancer MDTs is contributing to higher treatment use and improved outcomes for people diagnosed with lung cancer in major cities.^3,17^

Overall, one quarter of people had an emergency admission for lung cancer in the lead-up to diagnosis. More than half of these did not have GP-ordered imaging or a lung specialist attendance. A higher proportion of people who had emergency hospitalisations had lung cancer with distant spread compared with those without an emergency admission, suggesting that symptoms of late-stage disease are leading to emergency presentations in some cases. Lung cancer symptoms requiring emergency treatment can occur with little or no prior warning or after a prolonged period without the patient seeking help for milder symptoms.^18^ While community awareness programs aim to increase recognition of symptoms and encourage people to seek medical advice, there is evidence that people delay seeking medical attention for reasons including a perceived lack of urgency about symptoms and stigma associated with lung pathologies if they have been smokers.^19^ Another reason for emergency presentation could be referral to an emergency department, either by a physician or patient self-referral, as a way to avoid real or perceived waiting time, financial or distance barriers to accessing a lung specialist or diagnostic procedures in the community.^20,21^

It is difficult to compare our pathway results with other studies because of differences in data sources, methods and health systems. The proportion of people diagnosed following an emergency presentation in this study (25%) is lower than the figure of around one third reported in two other Australian studies and 39% in a UK study but much higher than the 6% in a Danish study.^9,22–24^ Although NSW emergency department data contains coded diagnostic information, these codes often reflect the presenting symptoms rather than the underlying diagnosis. The emergency department codes are assigned by a range of emergency department staff, whereas diagnoses in the NSW hospital data are coded by specialist medical coders using the medical record for each admission. Since people presenting to emergency departments who are diagnosed with lung cancer are likely to be admitted, we defined an emergency diagnosis pathway as one with an emergency hospital admission with a lung cancer diagnosis in order to avoid capturing people presenting to emergency departments who do not receive a definitive diagnosis. Another difference between studies is the definition of lung specialist. In the absence of information on lung cancer MDT membership, we included respiratory physicians and cardiothoracic surgeons, although some studies also include medical and radiation oncologists as lung specialists.^4,5,7^

A limitation of the data is that they do not contain presenting symptoms at GP and specialist attendances or imaging results. This meant that we were unable to calculate time intervals between first presentation with lung symptoms and diagnosis or treatment, assess the appropriateness of the observed pathways compared to a clinical guideline or standard, or identify people diagnosed incidentally. Although in our study health service use was similar across cities and rural areas in the lead-up to diagnosis, there may be disparity in the time between first presentation with symptoms and accessing specialist and diagnostic services that we could not measure. We were unable to identify a diagnostic procedure for around a third of people with a histopathological diagnosis, consistent with known under-reporting of diagnostic procedures in the hospital admission data.^25^

Smoking prevalence is lower among 45 and Up Study participants and smoking is the most important lifestyle risk factor in people presenting with lung cancer.^19,26^ 45 and Up Study participants diagnosed with lung cancer were less socio-economically disadvantaged, healthier and less likely to live in major cities than all NSW residents aged ≥45 years diagnosed with lung cancer.^27^ Hospital and emergency department use in the year prior to lung cancer diagnosis were similar between 45 and Up Study participants and all NSW residents; however, primary and outpatient care use could not be assessed.^27^ Participants of cohort studies may differ from non-responders in their health literacy and health-seeking behaviours. This may result in differences in diagnostic pathways compared with the general NSW population with lung cancer.

Studies of diagnostic pathways are often based on patient medical record reviews, surveys or interviews and are restricted to small geographic areas or a limited number of hospitals.^9–11^ A strength of this study is that linkage of health-related records for 45 and Up Study participants enabled the examination of the use of primary care, outpatient imaging, specialist care and admissions to hospital in the lead-up to diagnosis of lung cancer for a NSW-wide sample using data with population-level coverage, which had previously been a gap in understanding care pathways for lung cancer.

The Australian Optimal Care Pathway (endorsed in 2015) lays out expected pathways of initial investigations, referral, diagnosis and treatment for people presenting with suspected lung cancer with the aim of promoting best practice care regardless of where people live or have treatment.^28^ Our study focussed on GP-ordered imaging and specialist referral, which are steps laid out in the Optimal Care Pathway. The optimal pathway for a person with suspected lung cancer will depend on their presenting signs and symptoms and may include GP-referred imaging, referral to a specialist who is part of an MDT and referral of those with massive haemoptysis or stridor to the emergency department. Our study period is before the publication of the Optimal Care Pathway but can provide baseline data on health service use. Some areas in NSW have implemented localised optimal care pathways using GP decision aid software that provides management guidelines for people presenting with respiratory symptoms and referral details for lung specialists who are MDT members and for rapid access clinics. This implementation at a local level should assist with ensuring that people presenting with symptoms receive appropriate investigations in a timely way.

This study revealed that more than half of people did not attend a lung specialist in the diagnostic pathway of NSCLC. A quarter of people diagnosed presented as emergencies and more than half of those had no prior evidence of GP or lung specialist involvement in their diagnostic work-up. This study also highlights the key role that GPs have in the diagnosis of lung cancer. Ensuring that GPs have information available to them to initiate appropriate investigations and referral to specialists who are members of lung cancer MDTs has the potential to promote best practice care for people with lung cancer. Further research on barriers to recognition of lung cancer symptoms and access to lung specialists is needed from both a patient and physician perspective.

## Methods

### Design and ethical considerations

This descriptive study used linked population-based, health-related data sets for participants of the 45 and Up Study. Ethical approval was from the NSW Population and Health Services Research Ethics Committee (HREC/14/CIPHS/60). The 45 and Up Study was approved by the University of New South Wales Human Research Ethics Committee.

### Study cohort

Our study cohort was selected from participants of the Sax Institute’s 45 and Up Study.^29^ Participants were randomly sampled from the Department of Human Services (formerly Medicare Australia) enrolment database, which is a universal health-care scheme for citizens and permanent residents. Around 267,000 people joined the 45 and Up Study between February 2006 and December 2009 (18% participation rate) by completing a questionnaire and providing signed informed consent to follow-up and linkage of health records.

Our analysis cohort included people diagnosed with NSCLC (see Supplementary Table 1 for definition) between enrolment into the 45 and Up Study and 31 December 2012. The focus is on NSCLC because it is the most common type of lung cancer and less common histology types can have different presentations and outcomes. People with another cancer case diagnosed between 2000 and 2012, a NSCLC notified by death certificate only or with an unknown date of diagnosis were excluded.

### Data sources

Linkage of 45 and Up Study baseline questionnaire data to population-based health-related data sets was used to identify cancer diagnoses and health service use. Cancer case data were obtained from the NSW Cancer Registry, a statutory registry of all invasive cancer cases (excluding non-melanoma skin cancer) diagnosed in NSW residents. Hospital admission records for all NSW public and private hospitals were obtained from the NSW Admitted Patient Data Collection, which contains coded diagnostic and procedure information for each admission. GP attendances, specialist attendances, diagnostic imaging and diagnostic procedures performed in a community or outpatient setting were identified from Medicare Benefits Schedule claims data, which is a universal scheme providing subsidised medical services.

Linkage of the 45 and Up Study cohort to cancer and hospital data was performed by the Centre for Health Record Linkage (http://www.cherel.org.au) and to the Medicare Benefits Schedule data by the Sax Institute using a unique identifier provided to the Department of Human Services.

### Cohort characteristics

Demographic and cancer case data were obtained from the NSW Cancer Registry, including: date of diagnosis, most definitive method of diagnosis, extent of disease, age at diagnosis, sex, remoteness of residence (Accessibility/Remoteness Index of Australia [ARIA+]), and area-based socioeconomic disadvantage (Index of Relative Socioeconomic Disadvantage).^30,31^ Smoking status was obtained from the 45 and Up Study baseline questionnaire. The Charlson comorbidity score, excluding metastatic cancer, was calculated using hospital admissions in the 5 years prior to diagnosis.^32^

### Use of health services in the lead-up to diagnosis

We quantified the use of GP attendances, specialist attendances, chest X-rays and chest CT scans in a community setting (outside a hospital admission), diagnostic procedures (bronchoscopy or biopsy) in an outpatient or inpatient setting and hospital admissions related to the diagnosis of lung cancer in the 15 months prior to diagnosis of NSCLC (see Supplementary Table 1 for definitions). We calculated the proportion of people using each service in the lead-up to diagnosis, which we defined as the 3 months (91 days) up to and including the date of diagnosis, based on the study data and the findings of other studies.^12,21^ The date of diagnosis on the NSWCR during the study period is the date of histological or cytological confirmation for the majority of cases or, for those with a clinical diagnosis, the date of first hospital admission or outpatient consultation.

### Pathways to diagnosis

We defined pathways to diagnosis by the presence or absence in the lead-up to diagnosis of:

- GP-ordered chest imaging (X-ray or CT scan);
- lung specialist (respiratory physician or cardiothoracic surgeon) attendance in a community/outpatient setting;
- hospital admission with a lung cancer indication, by urgency of first admission (planned or emergency).

These events indicate the involvement of GPs, lung specialists and hospitals in the diagnostic work-up. GP attendances were not included in the diagnosis pathway analyses because we could not distinguish lung cancer-related attendances from attendances for unrelated conditions.

### Statistical analysis

We used Pearson’s chi-square tests to assess associations between remoteness of residence and use of health services, and between outcomes of interest (seeing a lung specialist and emergency presentation) and patient characteristics or other events on the diagnostic pathway. Two-sided *P* values are reported. Analyses were performed using SAS Enterprise Guide v6.1.

## Supplementary information

Supplementary Information

## Data Availability

The raw data that support the findings of this study are available from the Sax Institute, NSW Ministry of Health and Cancer Institute NSW. Restrictions apply to the availability of these data, which were used under license for the current study, and so they are not publicly available. Data are, however, available from the custodians of the data sources by following the 45 and Up Study (https://www.saxinstitute.org.au/our-work/45-up-study/for-researchers) and Centre for Health Record Linkage (www.cherel.org.au/apply-for-linked-data) application processes. Derived data are available from the authors upon reasonable request and with permission of the data custodians.

## Supplementary information

**Supplementary information** accompanies the paper on the *npj Primary Care Respiratory Medicine* website (10.1038/s41533-018-0113-7).
