# Supplementary material for: Pathways to diagnosis of non-small cell lung cancer: a descriptive cohort study
Source: NPJ Prim Care Respir Med. 2019 Feb 8;29:2. doi: 10.1038/s41533-018-0113-7 (PMC6368611; doi:10.1038/s41533-018-0113-7)
Supplement: Supplementary file 1 — Supplementary Information [file 41533_2018_113_MOESM1_ESM.pdf]

Supplementary Information

Supplementary Table 1. International Classification of Disease for Oncology 3rd Edition (ICD-O-3) codes, Medicare Benefits Schedule (MBS) item numbers and Australian Classification of Health Interventions (ACHI) 4th - 7th edition codes for the study definitions.

Area	Category	Dataset	Definition
**Cancer cases**	Non-small cell lung cancer (NSCLC)	NSWCR[1]	Site (ICD-O-3) C34.x and morphology (ICD-O-3):<br>8000-8941 excluding:<br>8040-8045 (small-cell lung cancers)<br>8240-8245 and 8249 (other lung cancers)
**Attendances**	GP attendance	MBS	MBS item number:<br>1-51 (GP attendances, other)<br>597, 599, 601, 602 (GP urgent after hours)<br>700-715 (Health assessments)<br>2497-2559 (GP attendances with PIP incentive)<br>5000-5067 (GP after hours, other)
	Specialist attendance	MBS	MBS item number:<br>99-109<br>113
	Consultant physician attendance	MBS	MBS item number:<br>110-112<br>114-133
**Specialist type**	Respiratory physician	MBS	Provider specialty code:<br>44 (CP-THORAC-MED)<br>118 (SP-THORAC-MED)<br>099 (THORACIC MEDICINE)
	Cardiothoracic surgeon	MBS	Provider specialty code:<br>8 (CARD-THR-SURG)<br>010 (CARDIO-THORACIC SURGERY)
	Medical oncologist	MBS	Provider specialty code:<br>33 (CP-MED- ONCLGY)<br>046 (MEDICAL ONCOLOGY)
	Radiation oncologist	MBS	Provider specialty code:<br>102 (RAD-ONCLGY-SP)<br>086 (RADIOLOGY ONCOLOGY-SPECIALIST)
	General physician	MBS	Provider specialty code:<br>28 (CP-GEN-MED)<br>108 (SP-GEN-MED)<br>035 (GENERAL MEDICINE)
	General surgeon	MBS	Provider specialty code:<br>21 (COL-TR-SURGEO)<br>56 (GEN-SRG-NOSTD)<br>57 (GEN-SURGERY)<br>036 (GENERAL SURGERY)<br>037 (GENERAL SURGERY, NON-STANDARD)<br>097 (SURGERY-TRAINEE)
**Imaging**	Chest X-ray	MBS	MBS item number:<br>58500-58508
	Chest CT scan	MBS	MBS item number:<br>56301-56347 Chest and upper abdomen<br>56801-56847 Chest, abdomen, pelvis and neck<br>57001-57047 Brain, chest and upper abdomen

Area	Category	Dataset	Definition
**Diagnostic procedures**	Bronchoscopy	APDC[2]	ACHI procedure code:<br>41889-00 Bronchoscopy<br>41889-01 Bronchoscopy through artificial stoma<br>41892-00 Bronchoscopy with biopsy<br>41898-00 Fibreoptic bronchoscopy<br>41898-01 Fibreoptic bronchoscopy with biopsy
		MBS	MBS item number:<br>30710 Endobronchial ultrasound-guided biopsy<br>41889 Bronchoscopy as an independent procedure<br>41892 Bronchoscopy with biopsy or other procedure<br>41898 Fibreoptic bronchoscopy with biopsy
	Other chest endoscopy	APDC[2]	ACHI procedure code:<br>38436-00 Thoracoscopy (incl. biopsy)<br>38448-01 Mediastinoscopy (incl. biopsy)
		MBS	MBS item number:<br>38436 Thoracoscopy, with or without division of pleural adhesions, including insertion of intercostal catheter where necessary, with or without biopsy<br>38448 Mediastinum, cervical exploration of, with or without biopsy
	Needle biopsy	APDC[2]	ACHI procedure code:<br>38812-00 Percutaneous needle biopsy of lung<br>30090-00 Percutaneous needle biopsy of pleura
		MBS	MBS item number:<br>30696 Endoscopic ultrasound guided fine needle aspiration biopsy(s) (endoscopy with ultrasound imaging) to obtain one or more specimens from either (a) mediastinal mass(es) or (b) locoregional nodes to stage non-small cell lung carcinoma<br>38812 Percutaneous needle biopsy of lung
	Open biopsy	APDC[2]	ACHI procedure code:<br>38418-02 Biopsy of lung (excl. endoscopic/percutaneous needle biopsy)
**Surgical treatments**		APDC[2]	ACHI procedure code:<br>38438-00 Segmental resection of lung<br>38438-01 Lobectomy of lung<br>38438-02 Pneumonectomy<br>38440-00 Wedge resection of lung<br>38440-01 Radical wedge resection of lung<br>38441-00 Radical lobectomy<br>38441-01 Radical pneumonectomy<br>41901-00 Endoscopic resection of bronchus by laser<br>90169-00 Endoscopic wedge resection of lung<br>41892-01 Bronchoscopy with excision of lesion<br>90163-00 Other endoscopic excision of bronchus

1. NSWCR = New South Wales Cancer Registry.
2. APDC = Admitted Patient Data Collection.
